# A comparison of cervical delta-shaped anastomosis and circular stapled anastomosis after esophagectomy

**DOI:** 10.1186/s12957-017-1097-4

**Published:** 2017-01-19

**Authors:** Chen Huang, Xunhai Xu, Binbin Zhuang, Wenshu Chen, Xunyu Xu, Chao Wang, Shengmei Lin

**Affiliations:** 1 Department of Thoracic Surgery, Fujian Provincial Hospital, Provincial Clinical College of Fujian Medical University, No. 134 East St, Fuzhou, 350001 Fujian China; 2 Gastrointestinal Endoscopy Center, Fujian Provincial Hospital, Fuzhou, 35001 China; 3 Department of Radiology, Fujian Provincial Hospital, Fuzhou, 350001 China

**Keywords:** Esophageal cancers, Esophagogastric anastomosis, Anastomotic leakage, Stenosis

## Abstract

**Background:**

The delta-shaped anastomosis has been reported to reduce anastomotic complications for a decade. However, little has been written comparing this technique with the circular stapler technique. The objective of this retrospective study was to assess the safety and efficacy of cervical delta-shaped anastomosis after esophagectomy.

**Methods:**

Medical records of patients with esophageal squamous cell carcinoma who underwent McKeown (three-incision) esophagectomy between September 2013 and June 2015 were reviewed. Either circular stapled anastomosis (CSA) or delta-shaped anastomosis (DSA) was performed at the cervical stage. The clinical characteristics and short-term outcome were retrospectively assessed to identify the differences between the two groups.

**Results:**

A total of 81 patients were included in this study. The clinical characteristics were similar between the two groups. Cervical anastomotic leakage occurred in 3 (7.7%) of 39 patients in the DSA group and in 8 (19%) of 42 patients in the CSA group (*P* = 0.197). The average anastomotic orifice width was 16.1 ± 4.9 mm and 11.7 ± 2.2 mm, respectively (*P* < 0.001). The incidence of anastomotic stenosis was 2.6% (1/39) and 23.5% (10/42) in the DSA and CSA groups, respectively (*P* = 0.007). There was no significant difference in surgical duration, blood loss, pulmonary complication, postoperative mortality, time of hospitalisation and time of ICU stay between the two groups.

**Conclusions:**

Delta-shaped anastomosis may be an effective alternative method for gastroesophageal anastomosis after esophagectomy, with lower incidence of leakage and stenosis.

## Background

Subtotal esophagectomy and reconstruction using gastric tube is considered the gold standard for surgical treatment of surgically resectable esophageal cancer (EC) [1]. However, anastomotic leakage and stricture formation continue to be major challenges after resection of EC. They are associated with high mortality and frequently compromise patient quality of life [2, 3].

Much effort has been devoted to reducing anastomotic complications in the most recent decade especially in anastomotic technique. With the advent of smaller endoscopic staplers, it was reported by several authors that cervical linear stapler anastomosis was associated with a lower rate of anastomotic complications [4–12].

Okushiba et al. [13] described a new technique of anastomosis named the ‘esophageal delta-shaped anastomosis’ in 2005. In his report, there were no complications associated with the anastomosis in all nine patients treated with the delta-shaped anastomosis. Such technique appears to result in a larger lumen size, shorter operative time, better blood supply, and less anastomosis margin tension. However, as far as we know, little has been reported comparing the results of delta-shaped anastomosis (DSA) to circular stapled anastomosis (CSA). In this article, we describe our surgical procedure using the DSA approach to create the cervical anastomosis and examine its efficacy and safety in comparison to CSA in patients undergoing esophagectomy for EC.

## Methods

### Patients

Medical records of consecutive patients with primary EC who underwent esophagectomy at Fujian Provincial Hospital between September 2013 and June 2015 were retrospectively reviewed. Patients who did not undergo McKeown (three-incision) esophagectomy with cervical esophagogastric anastomosis (CEGA) were excluded. The study was approved by the hospital ethics committee, and a waiver for individual patient consent for this retrospective study was also obtained from the ethics committee. All patients were diagnosed as esophageal squamous cell carcinoma by endoscopic biopsy. Preoperative physical examination, standard laboratory tests, and cardiac and pulmonary function test were performed. Contrast-enhanced thoracic computed tomography (CT) and abdominal CT were performed for preoperative staging to confirm disease status. Those patients with locally advanced EC received neoadjuvant chemotherapy prior to surgical resection. All operations were performed by the same surgeon (Xunyu Xu). The clinical characteristics of patients are shown in Table 1.

**Table 1:** Comparison of clinical characteristics between groups

Characteristics	CSA (*n* = 42)	DSA (*n* = 39)	Statistics	*P* value
Age	57.9 ± 8.2	61.0 ± 8.9	*t* = 1.386	0.171
Sex (male/female)	26/16	30/9	*χ* ^2^ = 2.138	0.158
BMI	21.5 ± 3.5	22.8 ± 3.3	*t* = −1.424	0.159
Diabetes	2 (4.8)	0 (0)		0.494^a^
Hypertension	5 (11.9)	5 (12.8)		1 ^a^
Coronary artery disease	2 (4.8)	2 (5.1)		1 ^a^
Pathologic stage				
I	12 (28.6)	13 (33.3)		0.784 ^a^
II	16 (38.1)	12 (30.8)		
III	14 (33.3)	13 (33.3)		
IV	0 (0)	1 (2.6)		
Location of tumour				
Upper	12 (28.6)	6 (15.4)		0.383 ^a^
Middle	24 (57.1)	27 (69.2)		
Lower	6 (14.3)	6 (15.4)		
Surgical approach				
MIE	24 (57.1)	28 (71.8)	*χ* ^2^ = 1.889	0.246
Open	18 (42.9)	11 (28.2)		
Neoadjuvant chemotherapy	7 (16.7)	5 (12.8)	*χ* ^2^ = 0.152	0.762

All values in parentheses denote the percentage

*BMI* body mass index (kg/m^2^), *MIE* minimally invasive esophagectomy

^a^Analysed by Fisher’s exact test

### Surgical techniques

A 3.0-cm-wide gastric tube was constructed along the lesser curvature of the stomach using linear staples (TLC75, Ethicon Endosurgery, Cincinnati, OH, USA) with seromuscular sutures along the staple line. The gastric tube was then pulled up through the posterior mediastinal route and extracted from the left neck incision for alimentary tract reconstruction. Fast-frozen section pathology was routinely performed to ensure a negative surgical margin. For CEGA, CSA was routinely performed until June 2014. After that time, CSA was applied only when the remnant esophagus was not long enough to perform DSA.

For DSA, after apposition of the posterior walls of the esophagus to the gastric tube, a 1-cm gastrostomy was created near the greater curvature in the posterior wall where the left gastroepiploic artery enters, because the region has a good blood supply and gives no extra tension.

**Fig. 1:**
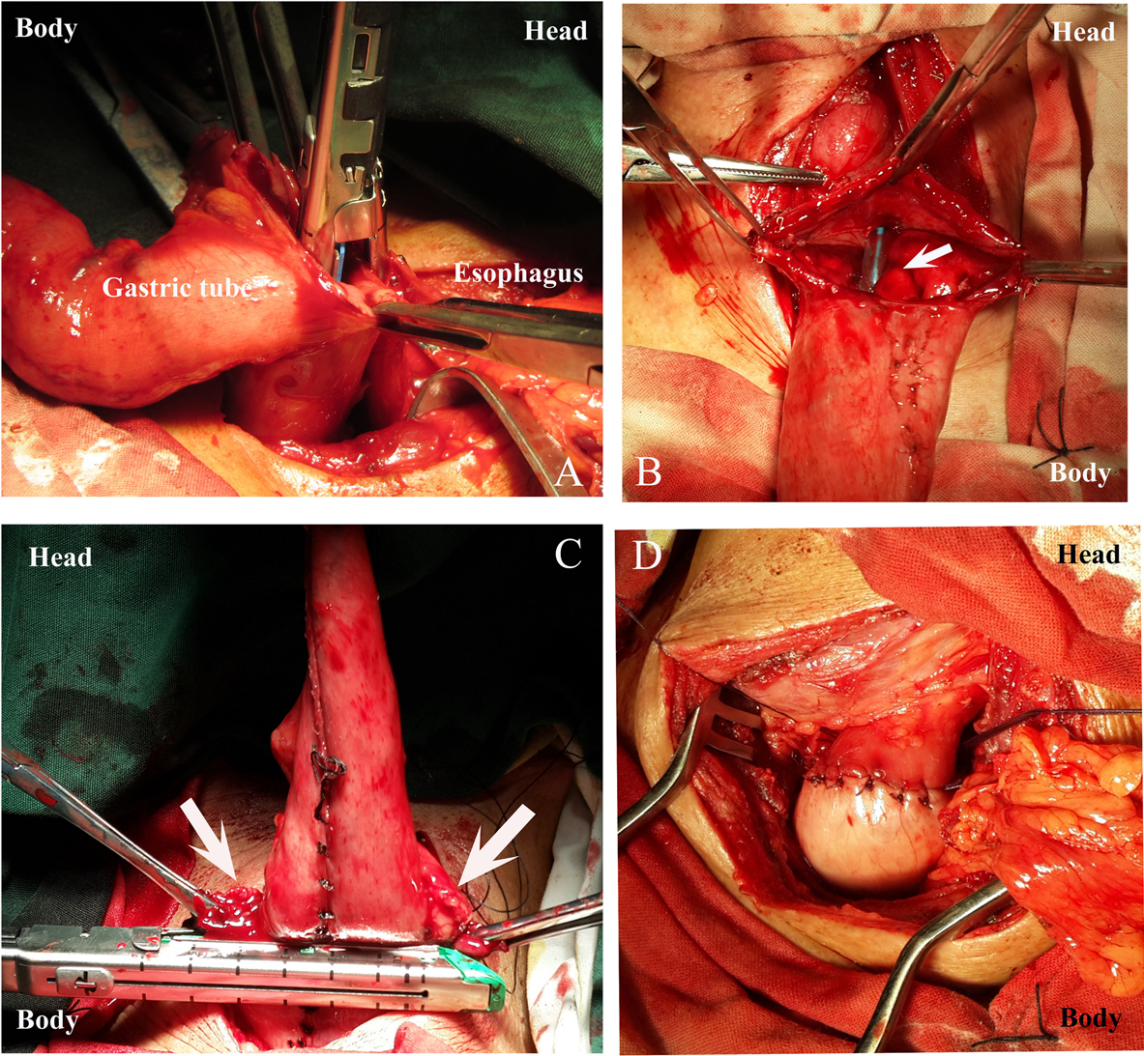
**a** The gastric tube was pulled up to the left neck incision, with apposition of posterior walls of the esophagus and gastric tube; a 1-cm gastrostomy was created at the posterior wall of the gastric tube. The anvil was placed in the remnant esophagus, and the staple cartridge was placed in the gastric tube. **b** After firing and removing the first linear stapler, a nasogastric tube (*white arrow*) was inserted and advanced downward into the gastric tube. The common opening in the stomach and esophagus were then grasped with Allis clamps and placed together for closure. **c** The second 60-mm linear staplers were fired transversely in an everted fashion. The common opening (*white arrows*) was closed and the tip of the gastric tube was excised. **d** The staple line was reinforced by interrupted serosal sutures with 4-0 absorbable Vicryl antibacterial sutures. The cervical DSA was completed

An endoscopic linear cutting stapler (EndoGIA60 mm-3.5 mm, Covidien, Mansfield, MA, USA) was used to create the posterior wall in an inverted fashion. The anvil was placed in the remaining esophagus and the staple cartridge was placed in the gastric tube (Fig. 1a). After firing and removing the linear stapler, a 14-French or 16-French nasogastric tube was inserted by the anaesthetist and advanced downward into the gastric tube. The common opening in the stomach and esophagus was then joined with Allis clamps and placed together for closure (Fig. 1b). The second and the third 60-mm linear staplers were fired transversely in an everted fashion (Fig. 1c). The common opening was subsequently closed and the tip of the gastric tube was excised. At last, the staple line was reinforced by interrupted serosal sutures with 4-0 absorbable Vicryl antibacterial suture (Polyglactin 910) (Fig. 1d). A closed suction drain was placed in the anastomotic region.

For CSA, a 25-mm circular stapler (Premium Plus CEEA AutoSuture, Covidien, Mansfield, MA, USA) was placed at the tip of the gastric tube, and the anvil of the stapler was inserted through the remaining part of the esophagus. Then an end-to-side esophagogastric anastomosis was performed. The tip of the gastric tube was excised by the linear staple (TLC75, Ethicon Endosurgery, Cincinnati, OH, USA). The staple line was subsequently reinforced by interrupted serosal sutures with 4-0 absorbable Vicryl antibacterial suture (Polyglactin 910).

### Definitions of anastomotic leakage and stenosis

A contrast swallow fluoroscopy on postoperative day 7 was obtained routinely to make sure whether anastomotic leakage happened. Oral intake would begin if the anastomotic leak was absent; otherwise, it would be delayed. The draining fluid and cervical incision sites were regularly inspected. In addition, the clinical status of the patients was also closely monitored to detect any sign of early anastomotic leakage. Anastomotic leakage was assessed based on four classified categories as proposed by the Surgical Infection Study Group [14]. Follow-up was conducted 3 months after surgery. The contrast swallow fluoroscopy and gastroscopy were performed to evaluate the anastomotic orifice. The width of the anastomotic orifice was measured by the same radiologist, who did not know the study design or anastomotic approach used. Anastomotic stenosis was defined as any stenosis in the anastomotic orifice, which required either endoscopic balloon dilation or bougienage at least once.

### Statistical analysis

Continuous data was reported as mean and standard deviation or median and range. Categorical data was reported as count and percentage. The chi-square test or Fisher’s exact test was used to analyse categorical data. Student’s *t* test was used to compare continuous variables. The Mann-Whitney *U* test was used for the unequal variances, such as time of intensive care unit (ICU) stay and width of anastomotic orifice. All tests were two-sided, and *P* values <0.05 were considered to indicate a statistically significant difference between the two groups. All of the statistical analyses were performed using the SPSS software package, version 17.0, for Windows (SPSS, Chicago, IL).

## Results

In total, there were 81 patients reviewed, including 56 males (69.1%) and 25 females (30.9%). The median age was 61 years (range, 38–76 years). There were 42 (51.9%) patients who underwent CSA and the other 39 (38.1%) patients underwent DSA. The clinical characteristics were similar between the two groups. Comparison of clinical characteristics between the two groups is shown in Table 1.

The surgical outcomes are shown in Table 2. There was a wider anastomotic orifice in DSA group compared to the CSA group (16.1 ± 4.9 mm vs. 11.7 ± 2.2 mm; *P* < 0.001). Anastomotic leaks tended to occur more frequently in the CSA group than the DSA group (19 vs. 7.7%; *P* = 0.197). Six patients in the CSA group and three patients in the DSA group had grade II anastomotic leakage, which were healed by cervical wound opening and drainage. Another two patients in the CSA group had grade I anastomotic leakage, and these were healed by delaying oral intake. There were no grade III–IV leaks in any of the patients. Three months after surgery, anastomotic stenosis developed significantly more frequently in the CSA group than the DSA group (23.8 vs. 2.6%; *P* = 0.007).

**Table 2:** Comparison of surgical outcome between groups

	CSA (*n* = 42)	DSA (*n* = 39)	Statistics	*P* value
Width of anastomotic orifice (mm)	11.7 ± 2.2	16.1 ± 4.9	*Z* = −4.057	<0.001
Anastomotic leak	8 (19.0)	3 (7.7)	*χ* ^2^ = 2.22	0.197
Anastomotic stenosis	10 (23.8)	1 (2.6)	*χ* ^2^ = 7.78	0.007
Pulmonary complication	20 (47.6)	17 (43.6)	*χ* ^2^ = 0.132	0.824
Perioperative mortality	0 (0)	2 (5.1)		0.229 ^a^
Surgical duration (min)	366.3 ± 60.0	360.7 ± 65.8	*t* = −0.337	0.737
Blood loss (mL)	385.4 ± 117.5	398.7 ± 179.7	*t* = 0.322	0.748
Mean duration of hospitalisation, days (range)	13.5 (12,75)	14 (10,76)	*t* = −1.551	0.126
Mean duration of ICU stay, days (range)	0 (0,6)	0 (0,27)	*Z* = −0.579	0.563

Unless otherwise stated, values in parentheses denote percentages

*ICU* intensive care unit

^a^Analysed by Fisher’s exact test

Perioperative mortality occurred in two patients in the DSA group (Adult Respiratory Distress Syndrome in one patient and aortic bleeding caused by tumour invasion in another patient). No perioperative mortality occurred in the CSA group, the difference did not reach statistical significance (5.1 vs. 0%; *P* = 0.229). There was no significant difference in surgical duration, blood loss, pulmonary complication, time of hospitalisation and time of ICU stay between the two groups.

## Discussion

Despite the improvement of anastomotic technique, CEGA leak after esophagectomy has been reported to occur in 15 to 25% of patients, which is an increased risk compared with chest anastomosis [3, 4]. One advantage of CEGA is that a CEGA leak is seldom associated with mediastinitis and is relatively benign and managed successfully with local wound care [4]. Cervical anastomotic leak is attributed to ischemia of the gastric conduit and the methods of anastomosis and surgical techniques [2]. The CSA technique has been introduced for nearly 30 years [15] and is considered to be useful for shortening operation time and reducing anastomotic leakage compared to hand-sewn anastomosis [16]. However, CSA results in end-to-side anastomosis, in which the circulation net of the gastric wall was blocked by the stapler, causing anastomosis failure [17]. The application of endoscopic linear stapler for CEGA has effectively reduced anastomotic complications compared to conventional hand-sewn anastomosis [4–6, 9]. Several studies showed that linear stapled technique had a lower frequency of anastomotic stenosis than CSA [8, 11, 18]. The DSA technique has been reported since 2005 [13]. It is a variation of modified Collard’s technique [5] in which the anterior wall of the esophagus and gastric tube is stapled. When DSA was performed, the first stapler line was parallel to the axis of the gastric tube, leading to maximum preservation of the vasculature network of the gastric tube. The linear stapler offered a triple-layered suture line which was more watertight than either a single- or double-layered hand-sewn anastomosis. Moreover, the tip of the gastric tube was excised in our technique, which improved the blood supply of the anastomotic orifice. All above are helpful to minimise anastomotic leak. In our study, a decrease of anastomotic leak was observed in the DSA group comparing it to the CSA group, although the difference did not reach statistical significance (19 vs. 7.7%; *P* = 0.197). There was no significant difference in surgical duration, blood loss, pulmonary complication, postoperative mortality, time of hospitalisation and time of ICU stay between the two groups. So DSA seems to be a safe and effective alternation for CEGA.

**Fig. 2:**
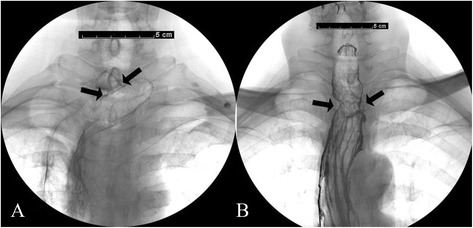
**a** A contrast swallow 3 months after CSA group operations. **b** A contrast swallow 3 months after DSA group operations. A wider anastomotic orifice (*black arrows*) and better passage in the DSA group is shown compared with the CSA group

**Fig. 3:**
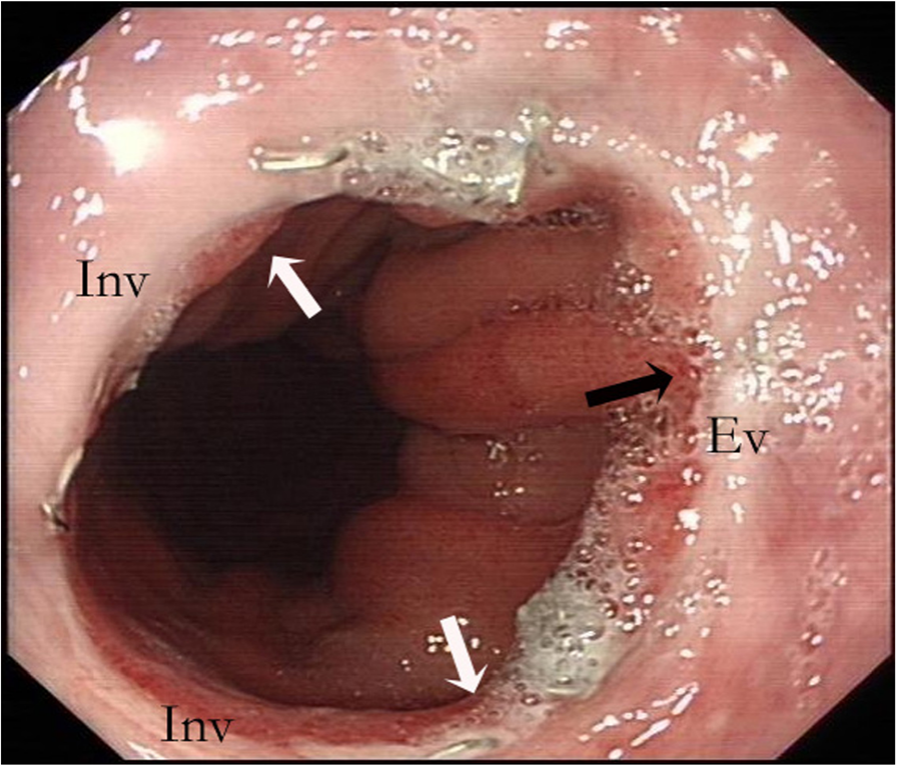
Endoscopic view of the anastomotic orifice 3 months after operation. The delta-shaped lumen is wide. The ‘V’ shaped posterior wall is inverted (*Inv*) (*white arrows*). The anterior wall is everted (*Ev*) so that there are no mucosal defects (*black arrow*)

Anastomotic stenosis is another serious anastomotic complication after esophagectomy, which is reported to occur in 27 to 45% of patients who underwent CSA [9, 19]. In CSA, the anastomotic lumen is dependent on the size of the esophagus and the replacement organ. Second, the CSA technique is an inverted anastomosis, creating a separate margin between the esophageal and gastric mucosa because they clamp the muscular tissues between them, resulting in anastomotic stricture from associated scar formation [18]. On the other hand, the DSA technique extended the anastomosis along the sidewall of the esophagus, thereby creating a larger lumen that is not affected by the size of the esophagus. In addition, this side-to-side technique performed on the posterior wall of the gastric tube and esophagus in effect created a functional end-to-end esophagogastric connection, which would be ideal for the passage of food (Fig. 2). In addition, the anterior wall of the anastomotic orifice was closed by two everted staples, which reduced the scar formation and anastomotic stenosis (Fig. 3). Specifically, we used a 60-mm linear stapler in the posterior wall to create a larger lumen size compared to Okushiba et al.’s [13] report. In our study, we observed a significant decrease of anastomotic stenosis (23.8 vs. 2.6%; *P* = 0.007) and a wider anastomotic orifice (16.1 ± 4.9 mm vs. 11.7 ± 2.2 mm; *P* < 0.001) in the DSA group compared with the CSA group.

However, DSA also has a limitation: it requires a longer proximal esophagus than CSA to be certain that a linear stapler can be placed. Therefore, it may be difficult in performing DSA when the tumour is too high in the upper esophagus. In our experience, at least a 5-cm remnant of the esophagus is needed; otherwise, we will select CSA for safe anastomosis.

Our results are limited by the nonrandomised retrospective design and smaller sample size, as well as the lack of exploration for long-term outcome of DSA. The result may be affected by the fewer upper esophageal tumours and more minimally invasive esophagectomy in the DSA group. We plan to conduct a randomised controlled study to further evaluate the DSA technique, including its short-term surgical outcome and long-term clinical benefits such as quality of life assessment.

In conclusion, our study directly assesses the short-term outcome between DSA and CSA. The preliminary data suggest our technique is a good alternative for gastroesophageal anastomosis because it creates a larger lumen size, which preserves and provides better blood supply. It appears to be a safe, effective, and straightforward surgical technique with improved short-term outcome, such as lower anastomotic leak and stenosis rate. We plan to conduct a randomised, controlled study with longer follow-up to assess the clinical benefit of this technique and draw a definitive conclusion.

## Conclusions

Delta-shaped anastomosis may be an effective alternative method for gastroesophageal anastomosis after esophagectomy, with lower incidence of leakage and stenosis.

## References

[1] Hochwald SN, Ben-David K (2012). Minimally invasive esophagectomy with cervical esophagogastric anastomosis. J Gastrointest Surg.

[2] Kassis ES, Kosinski AS, Ross P, Koppes KE, Donahue JM, Daniel VC (2013). Predictors of anastomotic leak after esophagectomy: an analysis of the society of thoracic surgeons general thoracic database. Ann Thorac Surg.

[3] Price TN, Nichols FC, Harmsen WS (2013). A comprehensive review of anastomotic technique in 432 esophagectomies. Ann Thorac Surg.

[4] Orringer MB, Marshall B, Iannettoni MD (2000). Eliminating the cervical esophagogastric anastomotic leak with a side-to-side stapled anastomosis. J Thorac Cardiovasc Surg.

[5] Ercan S, Rice TW, Murthy SC, Rybicki LA, Blackstone EH (2005). Does esophagogastric anastomotic technique influence the outcome of patients with esophageal cancer?. J Thorac Cardiovasc Surg.

[6] Behzadi A, Nichols FC, Cassivi SD, Deschamps C, Allen MS, Pairolero PC (2005). Esophagogastrectomy: the influence of stapled versus hand-sewn anastomosis on outcome. J Gastrointest Surg.

[7] Collard JM, Romagnoli R, Goncette L, Otte JB, Kestens PJ (1998). Terminalized semimechanical side-to-side suture technique for cervical esophagogastrostomy. Ann Thorac Surg.

[8] Li J, Shen Y, Tan L (2014). Cervical triangulating stapled anastomosis: technique and initial experience. J Thorac Dis.

[9] Toh Y, Sakaguchi Y, Ikeda O (2009). The triangulating stapling technique for cervical esophagogastric anastomosis after esophagectomy. Surg Today.

[10] Takemura M, Yoshida K, Fujiwara Y (2013). Modified triangulating stapling technique for esophagogastrostomy after esophagectomy for esophageal cancer. Surg Endosc.

[11] Xu QR, Wang KN, Wang WP, Zhang K, Chen LQ (2011). Linear stapled esophagogastrostomy is more effective than hand-sewn or circular stapler in prevention of anastomotic stricture: a comparative clinical study. J Gastrointest Surg.

[12] Noshiro H, Urata M, Ikeda O (2013). Triangulating stapling technique for esophagogastrostomy after minimally invasive esophagectomy. Surgery.

[13] Okushiba S, Kawarada Y, Shichinohe T, Manase H, Kitashiro S, Katoh H (2005). Esophageal delta-shaped anastomosis: a new method of stapled anastomosis for the cervical esophagus and digestive tract. Surg Today.

[14] Lerut T, Coosemans W, Decker G, De Leyn P, Nafteux P, van Raemdonck D (2002). Anastomotic complications after esophagectomy. Dig Surg.

[15] Liboni A, Mari C, Zamboni P (1989). A new technic for esophago-enteral anastomosis with a mechanical stapler without purse-string sutures. Ann Ital Chir.

[16] Kim RH, Takabe K (2010). Methods of esophagogastric anastomoses following esophagectomy for cancer: a systematic review. J Surg Oncol.

[17] Tabira Y, Sakaguchi T, Kuhara H, Teshima K, Tanaka M, Kawasuji M (2004). The width of a gastric tube has no impact on outcome after esophagectomy. Am J Surg.

[18] Wang WP, Gao Q, Wang KN, Shi H, Chen LQ (2013). A prospective randomized controlled trial of semi-mechanical versus hand-sewn or circular stapled esophagogastrostomy for prevention of anastomotic stricture. World J Surg.

[19] Furukawa Y, Hanyu N, Hirai K (2005). Usefulness of automatic triangular anastomosis for esophageal cancer surgery using a linear stapler (TA-30). Ann Thorac Cardiovasc Surg.

